# Gold Nanopeanuts as Prospective Support for Cisplatin in Glioblastoma Nano-Chemo-Radiotherapy

**DOI:** 10.3390/ijms21239082

**Published:** 2020-11-29

**Authors:** Joanna Depciuch, Justyna Miszczyk, Alexey Maximenko, Piotr M. Zielinski, Kamila Rawojć, Agnieszka Panek, Pawel Olko, Magdalena Parlinska-Wojtan

**Affiliations:** 1 Institute of Nuclear Physics Polish Academy of Sciences, 31-342 Krakow, Poland; justyna.miszczyk@ifj.edu.pl (J.M.); alexey.a.maximenko@gmail.com (A.M.); piotr.m.zielinski@ifj.edu.pl (P.M.Z.); agnieszka.panek@ifj.edu.pl (A.P.); pawel.olko@ifj.edu.pl (P.O.); magdalena.parlinska@ifj.edu.pl (M.P.-W.); 2 Nuclear Medicine Unit, Department of Endocrinology, The University Hospital, 31-501 Krakow, Poland; k.rawojc@gmail.com

**Keywords:** gold nanopeanuts, new shape, support, biomedical application

## Abstract

Herein, we propose newly designed and synthesized gold nanopeanuts (Au NPes) as supports for cisplatin (cPt) immobilization, dedicated to combined glioblastoma nano-chemo-radiotherapy. Au NPes offer a large active surface, which can be used for drugs immobilization. Transmission electron microscopy (TEM) revealed that the size of the synthesized Au NPes along the longitudinal axis is ~60 nm, while along the transverse axis ~20 nm. Raman, thermogravimetric analysis (TGA) and differential scanning calorimetry (DCS) measurements showed, that the created nanosystem is stable up to a temperature of 110 °C. MTT assay revealed, that the highest cell mortality was observed for cell lines subjected to nano-chemo-radiotherapy (20–55%). Hence, Au NPes with immobilized cPt (cPt@AuNPes) are a promising nanosystem to improve the therapeutic efficiency of combined nano-chemo-radiotherapy.

## 1. Introduction

Glioblastoma multiforme (GBM) is one of the most common and aggressive malignant brain tumors [1,2]. In GBM treatment, surgery, chemotherapy and radiotherapy are used [1]. Unfortunately, due to the location of tumor cells close to the brain and complex character of the tumor, categorized clinically into four different grades, GBM exhibits a poor prognosis and high rates of recurrence [1,3]. Radiation oncologists put in a lot of efforts to preferentially sensitize tumors to radiation, while minimizing effects in normal tissues and critical organs. One of the possibilities is the introduction of high-atomic number (Z) materials into the tumor cells [4]. One of the candidates can be gold, as it has a high atomic number and a high mass energy coefficient [5]. Moreover, the development of nanotechnology allows synthesizing differently shaped gold nanoparticles having such dimensions, which will allow them to accumulate in cancer tissue, due to the leaky intra-tumoral blood vessels and poorly formed intra-tumoral lymphatics [6,7,8,9,10,11]. Furthermore, gold nanoparticles are biocompatible, therefore they can be used as drug carriers [12]. Thus, a nanosystem can be created by immobilizing chemotherapeutic drugs molecules on the surface of gold nanoparticles (Au NPs), which can be used in combined nano-chemo-radiotherapy being a promising, revolutionary approach for the treatment of cancer [13,14]. The drugs, which are generally used in cancer treatment are cisplatin (cPt) or other platinate derivatives. The effects of cPt involve creating crosslinks between adjacent and within the same DNA strands. The formation of these crosslinks prevents DNA replication and cell division [15,16]. Unfortunately, chemotherapeutic drugs cause neurotoxicity, nephrotoxicity and ototoxicity effects in normal cells [17]. Over the years, different methods to reduce diverse side effects have been investigated [18,19]. The nanosystem (Au NPs + cPt) can be very effective, because its size will allow a direct delivery of the drug only to the cancer cells, which have a different morphology of blood vessels compared to healthy cells [20,21].

Currently, the most effective therapy against glioblastoma treatment is radiotherapy. The efficiency of this therapy is recognized as satisfactory, however, side effects are devastating for the organism, resulting in a poor condition of the patients after the treatment [22]. Therefore, scientists are working to continuously improve the success of radiotherapy with a simultaneous reduction of side effects, e.g., by combining it with conventional chemotherapy or, in recent years, with the addition of nanoparticles (NPs) like gold [23,24]. Consequently, herein we propose a combined nano-chemo-radiotherapy treatment for glioblastoma cell lines. For this purpose, we designed a new nanosystem consisting of gold nanopeanuts (Au NPes) with cisplatin immobilized on their surface, which will be added into the glioblastoma cells and subsequently treated by radiotherapy [25]. The peanut-like shape of the Au NPs was designed in a way to obtain a larger active surface but keep the cytotoxicity of the NPs as low as for spherical NPs.

## 2. Results

### 2.1. Characterization of NPs

**Figure 1:**
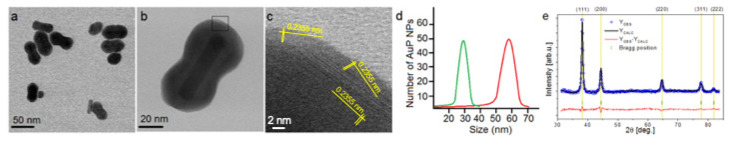
Overview BF STEM (**a**,**b**); HRSTEM image with indicated lattice distances of Au corresponding to the {111} planes. (**c**), size distribution of longitudinal (red curve) and transverse (green curve) axis (**d**) and XRD (**e**) taken for Au NPes. Experimental (blue dots) and calculated (solid black line) Rietveld refinement plot of the XRD pattern for the Au NPes. The red line (YOBS—YCALC) represents the difference between the observed and calculated data. Tick marks show allowed Bragg reflections (color online).

The bright field scanning transmission electron microscopy (BF STEM) image of the Au NPes is shown in Figure 1a. Indeed, the synthesized Au NPs have a peanut-like shape with nicely rounded edges, as expected in the design process. The size of the obtained NPs is ~60 nm along the longitudinal axis, while along the transverse axis ~20 nm. The information about the structure of the synthesized nanoparticles was acquired using X-ray diffraction (Figure 1d). The XRD pattern of the Au NPes was refined with the Rietveld method as shown in Figure 1d. Within the detection limit, all peaks could be attributed to standard Bragg reflections (111), (200), (220), (311) and (222) of Au nanocrystals with a face-centered cubic lattice [26,27], and the calculated cell parameter of the Au nanopeanuts was equal to 4.078 Å with uncertainties of ~0.001 Å. The average crystallite size of the Au NPes, estimated in the framework of micro-structural analysis from Fullprof, was around 18 nm and corresponded nicely to the size of Au NPes along the transverse axis determined by TEM.

### 2.2. Verification of Success of Functionalization and cPt Immobilization Process

**Figure 2:**
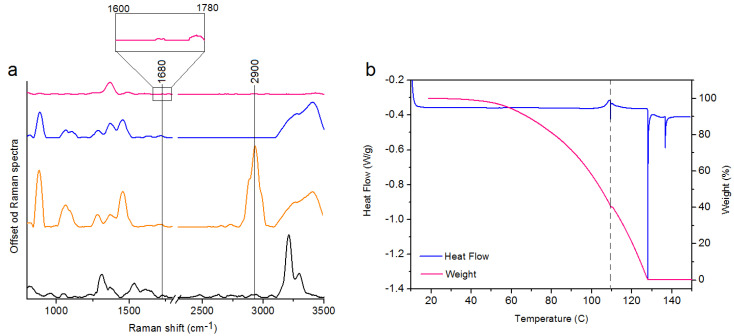
Raman spectra of: cPt (spectrum black), the pure MHDA (spectrum orange), the Au NPes + MHDA (spectrum blue), cPt@Au NPes (spectrum pink) (**a**); TGA and DSC data of cPt@Au NPes showing the solvent removal and decomposition of the product (**b**).

In this study, we show a successful functionalization of Au NPs, which is very important for an effective delivery of the drug to the cancer cells. Raman spectra of the four samples i.e., cisplatin (Figure 2a, black spectrum), the pure MHDA (Figure 2a, orange spectrum), and the cPt@Au NPes (Figure 2a, pink spectrum) were acquired. In the Raman spectrum of the MHDA sample, a peak of the thiol group (–SH) at 2900 cm^−1^ is visible [28]. The disappearance of this peak in the functionalized and cPt containing nanopeanut samples confirms a successful attachment of the MHDA to the surface of gold nanopeanuts. Moreover, in the Raman spectrum of cPt@Au NPes (Figure 2a, pink spectrum) a peak corresponding to the C = O vibrations (1680 cm^−1^) is observed [28]. This group is responsible for linking cPt with the functionalizing surfactants on the Au NPes surface [29]. Both observations provide the evidence on the success of Au NPes functionalization and cPt immobilization.

The results of TGA and DSC experiments, indicate that the cPt@Au NPes sample remains thermally stable up to 100 °C at a 5 °C min^−1^ heating rate, Figure 2b. The shape of the TGA signal points towards a strong evaporation of the solvent from the liquid sample visible between ca. 68 °C (5% weight loss) and 119 °C (59.7% weight loss), followed by its chemical decomposition from ca. 120 °C to 138 °C (40.03% weight loss). The DSC thermogram shows no signs of any significant thermal anomalies up to 110 °C. The anomalies registered above that temperature, as well as the baseline shift are effects of rapid boiling and decomposition of the sample or destabilization of MHDA [30]. However, in this experiment the stability of the nanosystem in the temperature range between 20 °C and 40 °C (room temperature–body temperature) is the most important.

### 2.3. Impact of Studied Nano-Chemo-Radiotherapy Approach on Cell Viability

**Figure 3:**
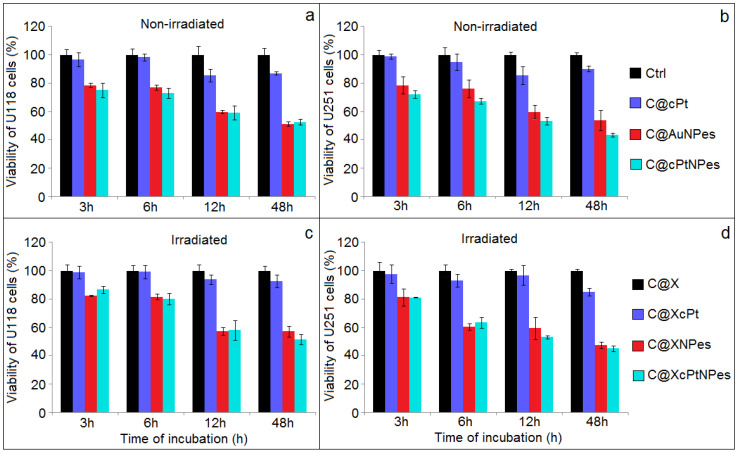
Comparison of the viability of U118 MG and U251 MG cell lines determined via the MTT assays in not-irradiated (**a**,**b**) and irradiated (**c**,**d**) cells by 2 Gy of X-rays after different time of incubation (3, 6, 24 and 48 h) with cisplatin, Au NPes and cPt@Au NPes and compared with controls.

Figure 3 shows the viability of the control and the non-irradiated U118 MG (Figure 3a) and U251 MG (Figure 3b) cell lines incubated with cPt, Au NPes or cPt@Au NPes for 3, 6, 24 and 48 h. The MTT assay showed a slight, but continuous decreasing in the viability of the treated cell lines with increasing incubation time for all investigated samples. The differences in viability observed between the two studied cell lines might be explained by the large degree of heterogeneity found in tumors [31]. The addition of cPt resulted in 1~15% mortality, depending on the cell line and incubation time. The most prominent effects in cell killing were observed for Au NPes and cPt@Au NPes. Studies show that nanoparticles can easily cross or interact with the cell membranes inducing antiproliferative activity and consequently cell death [32]. Interestingly, the addition of cPt@Au NPes caused a gradual decrease of cell viability, varying from 72–75% for 3h incubation to 44–53% for cell lines incubated for 48 h. However, significant differences between the addition of Au NPes vs. cPt@Au NPes were observed only for the U 251 MG cell line (*p* < 0.05). Au NPes coupled with cPt better inhibit the growth of GBM cells compared.free cisplatin and spherical Au NPs with attached CPt [33]. It could be due to the larger active surface of gold nanopeanuts compared to spherical Au NPs, which allow for the attachment of a higher density of cPt. Consequently, the local concentration of the drug in the tumor can be much higher, then when cPt is administrated intravenously. As already mentioned, the main advantage of the nanopeanuts is their shape, until now not described in literature. They can be compared to nanorods, which have sharper ends, and are thus more cytotoxic, than spherical Au NPs. In another work [34], the authors show that a concentration of 0.5 × 10^−6^ mg/mL is sufficient to cause a 20% mortality of cancer cells, while a concentration of 2.93 × 10^−6^ mg/mL of NPes has to be added to cancer cells to cause the same mortality effect. This is most probably due to the rounded ends of the nanopeanuts, having a similar shape to spherical nanoparticles, known for their lowest cytotoxic properties [35].

Irradiating with 2 Gy X-ray dose resulted in a gradual decrease of cell viability for both cell lines cultured with cPt, Au NPes or cPt@Au NPes as a function of incubation time after irradiation (Figure 3c,d). cPt@Au NPes were more efficient in inducing the U118 MG cell killing compared to Au NPes, which is evidenced by a significant decrease in the viability measured 48 h after irradiation (*p* < 0.05). Numerous factors, in addition to the genetic status mentioned above, have shown to influence the response of gliomas after radiotherapy [36]. The observed differences may be caused by inherent cellular radiation sensitivity between studied cell lines, the mechanism of radiation damage repair and the status of proliferation rates [37]. Independently on the culture additive (cPt, Au NPes or cPt@Au NPes), the cell mortality was higher with X-ray irradiation than without. Summarizing, for both cell lines the highest mortality was observed for combined nano-chemo-radiotherapy approach using the cPt immobilized on nanopeanuts nanosystem, rather than radiotherapy or chemotherapy alone. However, these differences are significant when we compare these results with C@XNPes cells.

Moreover, we also studied using FT-Raman and Fourier Transform InfraRed (FTIR) spectroscopy, the chemical changes in cells caused by cPt, Au NPes, cPt@Au NPes in combination with and without X-ray irradiation. The results of these experiments are presented in supplementary material (Figure S1–S3, Table S1).

## 3. Materials and Methods

### 3.1. Materials

Cetrimonium bromide (CTAB), chloroauric acid tetrahydrate (HAuCl_4_), silver nitrate (AgNO_3_), sodium borohydride (NaBH_4_), sodium citrate (Na_3_C_6_H_5_O_7_), L-ascorbic acid (C_6_H_8_O_6_), 16—mercaptohexadecanoic acid (MHDA), dimethylformamide (DMF), pentafluorophenyl (PFP), *N*, *N*diisopropylethylamine (DIPEA), *N*-cyclohexyl-*N*′-(2-morpholinoethyl) carbodiimide metho-p-toluenesulfonate (CMC) and all other chemicals were purchased from Sigma–Aldrich (Darmstadt, Germany).

### 3.2. Methods

#### 3.2.1. Au Nanorods Synthesis

##### Synthesis of Au Nanoseeds

0.364 g of CTAB was dissolved in 5 mL of water. Next, 5 mL of solution formed by dissolving 0.0017 g chloroauric acid tetrahydrate in 10 mL of water, were added. Finally, 0.6 mL of 100 × 10^−3^ M sodium borohydride was added to this solution. The solution was permanently under vigorous stirring. The reaction was stopped when the solution color changed to red.

##### Synthesis of Au Nanopeanuts

0.364 g of CTAB was dissolved in 5 mL of water. Next, 0.2 mL of solution formed by dissolving 0.0135 g silver nitrate in 20 mL of water, was added. Subsequently, 0.0017 g chloroauric acid tetrahydrate was dissolved in 5 mL of water and was added to the solution. Finally, 70 μL of Au nanoseed solution formed by dissolving 0.1386 g L-ascorbic acid in 10 mL of water and 30 μL of Au nanoseeds were added to the previous solution.

#### 3.2.2. Functionalization of Au NPes

**Figure 4:**
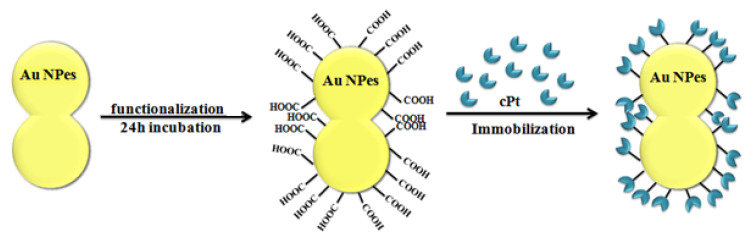
Scheme of the Au NPes functionalization process.

In order to attach the drugs to the stabilized Au NPes 5 mM 16—mercaptohexadecanoic acid (MHDA) was added into Au NPes solutions and was incubated overnight at 4 °C. After rinsing with dimethylformamide (DMF), the MHDA-covered Au-structures were incubated in DMF solution of 20 mM pentafluorophenyl (PFP) and 20 mM *N*, *N*diisopropylethylamine (DIPEA) and 20 mM *N*-cyclohexyl-*N*′-(2-morpholinoethyl) carbodiimide metho-p-toluenesulfonate (CMC), during 30 min at 25 °C. After repeated rinsing with DMF and centrifugation, the cisplatin solution was added and incubated for 30 min. at 25 °C. Consequently, th final concentration of cPt in the NPes surface was 1.71 × 10^3^ cPt molecules per one nanopeanut. In the immobilization process, an amide group is responsible for the coupling of COOH from MHDA with NH_3_ from cisplatin by covalent bond. The scheme of functionalization Au NPes is shown in Figure 4.

#### 3.2.3. TEM Characterization

The synthesized nanoparticles were imaged using transmission electron microscopy (TEM) using the high-angle annular dark field detector (HAADF) in the scanning mode. The observations were executed on an aberration-corrected FEI Titan electron microscope equipped with a FEG cathode working at 300 kV. To evaluate the particle size distribution, the diameter of 100 nanoparticles was measured on HRSTEM images acquired at different areas of TEM grids.

#### 3.2.4. X-ray Diffraction

The crystal microstructure of the Au NPes was assessed using a two-circle laboratory diffractometer Panalytical X’Pert Pro. The measurements were performed in the standard θ-2θ geometry using a lamp with the Cu anode working at 40 kV and 30 mA. The X-ray beam emitted by the X-ray tube was converted into a parallel beam by a divergence slit with a constant height 1/2°, a parabolic graded W/Si mirror with an equatorial divergence less than 0.05 and by a mask of constant width of 20 mm, limiting the width of the beam. The diffracted beam optics were composed of the anti-scatter slit with a height of 8.7 mm, 0.04 rad Soller slit collimator, a curved graphite monochromator to remove the contribution of the Cu Kβ radiation and the semiconductor silicon stripe detector with active length of 2.122°. The nanoparticles dispersion was dried on a zero-background holder and was mount on a sample spinner with rotation time of 16 s. The data were collected in the range between 20–80° (2θ) with a step size of 0.08°. The time per step was equal to 7000 sec. Fullprof software was used for diffractogram fitting, lattice constants and the coherent scattering length evaluation [38].

#### 3.2.5. Calorimetric Measurements

Calorimetric measurements were carried out with a TA Instruments’ DSC 2500 differential scanning calorimeter equipped with a liquid nitrogen LN2P pump. The samples (ca. 1–3 mg) were placed in aluminum pans and crimped with hermetic lids. The thermal behavior of the samples was studied under dry N5.0 pure nitrogen purge (25 mL min^−1^) in a temperature range from 10 °C to 200 °C at 5 and 10 °C min-1 heating rate and at 5 °C min^−1^ cooling rate. The samples were held isothermally at the minimum and maximum temperature for 5 min. Calibrations of temperature and enthalpy were performed using an indium standard. TRIOS software was used to calculate the peak temperatures and the enthalpy values of the registered thermal events. The TGA measurements were performed using a TA Instruments’ TGA 5500 thermogravimetric analyzer with high temperature platinum pans. The samples were either directly placed in open 100 µL platinum pans or previously enclosed inside hermetic aluminum pans to the limit the evaporation rate. In the case of aluminum pans, a hole of 0.9 mm diameter was punched through the lid of the container right before the start of the measurements. The samples were heated at a rate of 5 or 10 °C min-1 rate up to 500 °C under a flow of N5.0 pure nitrogen (25 mL min^−1^). The temperature calibration was carried out using nickel and aluminium tandards.

#### 3.2.6. Cell Lines

Two human glioblastoma cell lines (U118 MG, U251 MG) were used as an in vitro model. The U-118 MG was obtained from the ATCC^®^ (American Type Culture Collection, Virginia, No. HTB-15™) and was continuously cultured in Dulbecco’s modified Eagle’s medium (DMEM, ATCC^®^, No. 30-2002). The U251 MG (No. 09063001) human glioblastoma astrocytoma cell line was purchased from PHE (Public Health England, Salisbury, UK) culture collections and cultured in Dulbecco’s modified Eagle’s medium (DMEM, high glucose, GlutaMAX™, Gibco^®^, No. 31966021, Thermo Fisher Scientific, Waltham, MA, USA). The cells were maintained in 25 cm^2^ tissue culture flasks (TPP^®^, No. 90026, ) and the cultures were supplemented with 10% fetal bovine serum (FBS, Gibco^®^, No. 10270106), in a humidified incubator at 37 °C with 5% CO_2_. When 70% confluent cell monolayer was reached, the cells were detached with Trypsin/EDTA solution 1× (Sigma-Aldrich. No. T4049, Darmstadt, Germany) until complete cell detachment. The cell suspension with trypsin inactivated by a 5-fold dilution with fresh growth medium was spun down (3 min, 700 rpm) in 15 mL conical tubes, and the cell pellet was then resuspended in a fresh growth medium for cells scoring.

#### 3.2.7. Irradiation and Dosimetry

Single X-ray dose of 2 Gy was given to each dish or microplate (one dose per each plate containing a set of different samples) by a Philips X-ray machine (model MCN 323) at 250 kV, 10 mA, the dose rate of 2 Gy/min operating at the IFJ PAN. Detailed information about setup and dosimetry are provided in our previous studies [39]. Control (non-irradiated) and radiation plates were rinsed in a similar manner and kept out of the incubator (during radiation) for the same amount of time. After irradiation, all plates were immediately placed back to the incubator. Cell survival was assessed with the MTT assay and chemical changes were measured by spectroscopic experiments (see supplementary materials).

#### 3.2.8. Cell Viability Experiments

For MTT experiments, carried out in triplicates for each trial, 200 µl of suspension containing about 2500 cells of the U118 MG and U251 MG cell lines, respectively, were seeded per well in 96-well microplates (TPP^®^, No. 92096) and incubated for 72 h. A quarter of these cells was left without any further treatment and used as a control. From the rest of the 72 h incubated cells, the old medium was aspirated and cisplatin (5 uM) or Au NPes (2.93 × 10^−6^ mg/mL) or cPt@Au NPes (5 uM) were added to obtain a final volume of 110 µL per well. Similarly, both cell lines after 72 incubation and cells with the addition of cisplatin (5 uM) or Au NPes (2.93 × 10^−6^ mg/mL) or cPt@Au NPes (5 uM) were irradiated (see 2.8). The MTT tests were performed on all cells, including the reference cells, before and after irradiation, with and without additions after 3, 6, 24 and 48 h. The description of all cell samples, which were studied in MTT, Raman and FTIR experiments, is presented in Table 1.

#### 3.2.9. MTT Assay

The MTT reagent (10 µL, final concentration 0.5 mg/mL, Roche, Mannheim, Germany, No. 11465007001) was added to each well and incubated for the following 4 h at 37 °C. For solubilization of the resultant formazan product, 100 μL of the solubilization solution (10% *v*/*v* sodium dodecyl sulphate and 0.01 M HCl, Roche, Mannheim, Germany) was added to each well for overnight incubation. The absorbance at 590 nm was measured using a Tecan Spark microplate reader (Tecan Trading AG, Männedorf, Switzerland) [40].

#### 3.2.10. Analysis of Cell Viability Data

In the MTT test, data for each treatment point shows an average from three parallel wells with a standard deviation (±S.D.). For each well, five measurements at different positions were performed. To obtain pure cellular absorption, the absorption rate of the wells without cells was subtracted from the absorption of the wells with cells. According to the following equation based on Sadri A. [41] and explained by Zakrzewska K. [2], there is a direct association between the pure absorption and the rate of viable cells:

A_s/A_ref · 100% = v_c(1)

where A_s is the mean absorption of the measured sample, Aref is the mean absorption of the reference sample and v_c is the percentage of viable cells.

The data were analyzed and presented graphically using Microsoft Office Excel 2013. The quantitative results were finally compared with the *T* test. *P* value < 0.05 was considered to be statistically significant.

#### 3.2.11. Sample Preparation for Spectroscopic Experiments

In spectroscopic experiments, the U118 MG and U251 MG cells (125 × 10^5^ cells in a volume of 0.2 mL) were placed in 100 mm dishes (Sarstedt, No. 83.3902, Nümbrecht, Germany) and incubated for 72 h. The samples were prepared in the same way as for the MTT experiments. Then, the cells were detached, collected into 15 mL conical tubes, spun down (3 min, 700 rpm). The cell pellet was resuspended in 4 mL of 0.9% NaCl and once again spun down. The supernatant was carefully removed, and the pellets were measured by FTIR and Raman spectroscopy.

#### 3.2.12. FTIR Spectroscopy

EXCALIBUR FTS-3000 spectrometer working at room temperature was used for all measurements. All spectra were recorded by attenuated total reflection (ATR) with a ZnSn crystal. For that purpose, 0.5 mL of the cell containing solution was deposited on the ATR ZnSn crystal. The spectra were recorded in the range from 4000 to 500 cm^−1^. Each spectrum was obtained by averaging 64 scans recorded at a resolution of 4 cm^−1^. OPUS software was used for baseline correction and normalization of the obtained spectra.

#### 3.2.13. FT-Raman Spectroscopy

FT-Raman spectra were acquired with a Nicolet NXR 9650 FT-Raman Spectrometer equipped with an Nd:YAG laser (1064 nm) and a germanium detector. The measurements were performed in the between 150 and 3.700 cm^−1^ using laser power of 1 W. An unfocused laser beam was used with a diameter of approximately 100 μm and a spectral resolution of 8 cm^−1^. Omnic/Thermo Scientific software was used to process the Raman based on 128 scans.

## 4. Conclusions

In the present study the efficiency of combined nano-chemo-radiotherapy treatment for glioblastoma cell lines was examined. For this purpose, we designed a new nanosystem consisting of gold nanopeanuts (Au NPes) with surface immobilized cisplatin, which was added into the glioblastoma cells, which were subsequently subjected to X-ray radiation. TEM in combination with XRD revealed that the Au NPes are crystalline and their size is around 60 nm along the longitudinal axis, while it is ~20 nm along the transverse axis. The functionalization of the Au NPes as well as the immobilization success and stability of cPt on the Au NPes surface were verified by Raman spectroscopy. The TGA and DCS measurements showed that the created nanosystem is stable in the temperature range between 0 °C to 110 °C. The MTT assay showed that the highest mortality of cells was visible in the cells treated by combined therapy. Furthermore, FTIR and Raman spectra of cancer cells cultured with cisplatin immobilized on Au NPes surface and irradiated by X-rays showed the most visible chemical changes in the cells, especially in the functional groups building DNA, proteins and lipids compounds. Summarizing, the novel gold nanopeanuts are a promising support for delivering cPt into the glioblastoma cells and thus a promising nanosystem to improve the therapeutic efficiency of combined nano-chemo-radiotherapy.

## Figures and Tables

**Table 1:** Description of cells samples.

No.	Sample	Name of Samples in the MTT, Raman and FTIR Results
1	Control samples of cell lines U118 and U251 (cells without addition other substances)	Ctrl
2	Cells cultured with Au NPes	C@Au NPes
3	Cells cultured with 5 uM cisplatin	C@cPt
4	Cells cultured with Au NPes functionalized by cisplatin	C@cPtNPes
5	Control samples X-ray treated	C@X
6	Cells cultured with Au NPes and X-ray treated	C@XNPes
7	Cells cultured with 5uM cisplatin and X-ray treated	C@XcPt
8	Cells cultured with NPs functionalized by cisplatin and X-ray treated	C@XcPtNPes

## Supplementary Materials

Supplementary materials can be found at https://www.mdpi.com/1422-0067/21/23/9082/s1.
