# Supplementary material for: Gold Nanopeanuts as Prospective Support for Cisplatin in Glioblastoma Nano-Chemo-Radiotherapy
Source: Int J Mol Sci. 2020 Nov 29;21(23):9082. doi: 10.3390/ijms21239082 (PMC7730046; doi:10.3390/ijms21239082)
Supplement: Supplementary file 1 [file ijms-21-09082-s001.pdf]

Supplementary Materials:

Gold Nanopeanuts as Prospective Support for Cisplatin in Glioblastoma Nano-Chemo-Radiotherapy

Joanna Depciuch [1,*], Justyna Miszczyk [1], Alexey Maximenko [1], Piotr M. Zielinski [1], Kamila Rawojć [2], Agnieszka Panek [1], Pawel Olko [1,†] and Magdalena Parlinska-Wojtan [1,†]

[1] Institute of Nuclear Physics Polish Academy of Sciences, 31-342 Krakow, Poland; justyna.miszczyk@ifj.edu.pl (J.M.); alexey.a.maximenko@gmail.com (A.M.); piotr.m.zielinski@ifj.edu.pl (P.M.Z.); agnieszka.panek@ifj.edu.pl (A.P.); pawel.olko@ifj.edu.pl (P.O.); magdalena.parlinska@ifj.edu.pl (M.P.-W.)

[2] Department of Endocrinology, Nuclear Medicine Unit, The University Hospital, Krakow, Poland; k.rawojc@gmail.com

* Correspondence: joanna.depciuch@ifj.edu.pl

† Equal contribution as senior authors.

Chemical changes in the cells

In the Raman (Figure S1) and FTIR (Figure S2) spectra of all analyzed samples, peaks corresponding to functional groups building molecules of nucleic acid, polysaccharides, proteins and lipids functional groups, were observed. Moreover, the differences in the maximum absorbance values or Raman intensities, were visible, when we compare the investigated samples to the Ctrl. This observation shows the changes in the quantitative chemical compositions caused by Au NPes, cPt, cPt@Au NPes and X-ray irradiation. Furthermore, different positions of individual peaks in FTIR and Raman spectra depending on the sample, were noticed. The shifts of peaks suggest structural changes in the chemical compounds building functional groups. Moreover, their local chemical environments are different.

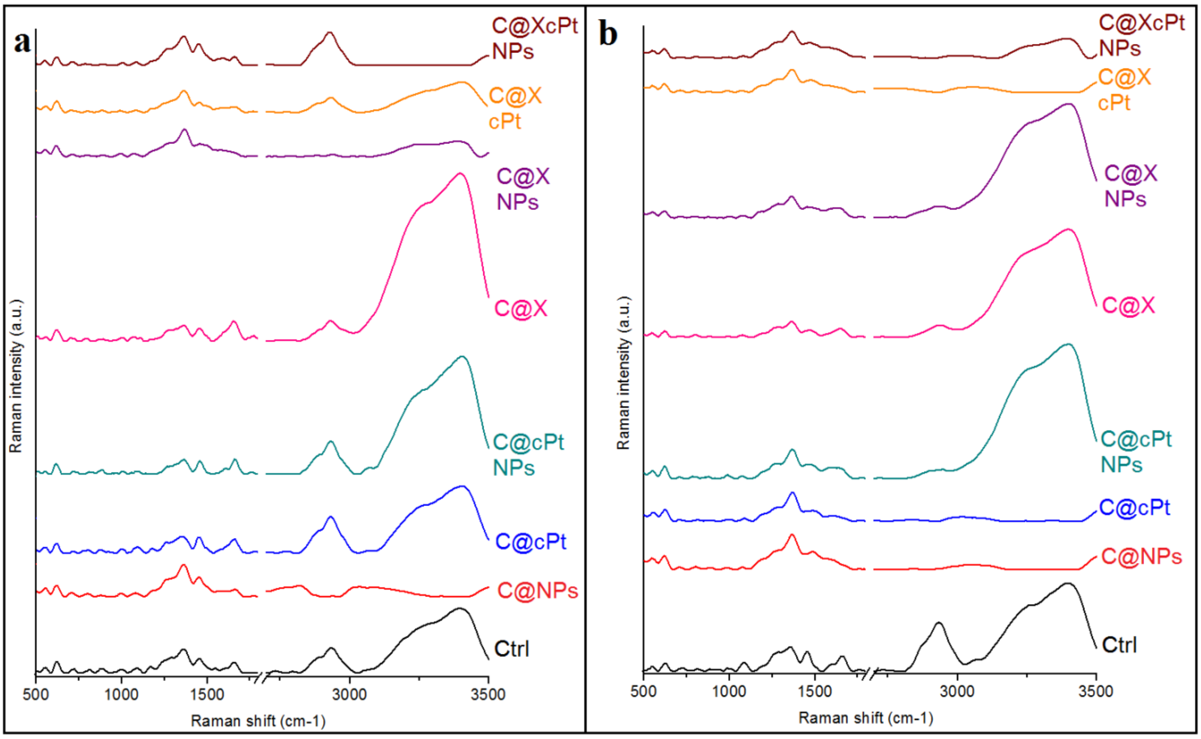

Figure S1. Raman spectra of U118 (**a**) and U251 (**b**) cells: Ctrl (black spectrum); C@NPs (red spectrum); C@cPt (blue spectrum); C@cPtNPs (green spectrum); C@X (pink spectrum); C@XNPs (violet spectrum); C@XcPt (orange spectrum); C@XcPtNPs (brown spectrum).

When we compare the investigated samples with the Ctrl, we observe significant changes in the structure of the chemical compounds, which are visible as shifts or absences of peaks. In the Raman spectrum of U118 C@NPs shifts of peaks corresponding to functional groups building glucose, lipids and amide I, as well as the absence of the peak at 1292 cm^{-1}, were observed. Moreover, cPt addition to cell culture caused structural changes in the amides II and III and phenylalanine. In the Raman spectrum of U118 C@cPtNPs absence of peaks at 995 cm^{-1}, 1089 cm^{-1} and 1548 cm^{-1}, as well as a shift of the peak originating from amide II vibrations, were visible. X-ray irradiation caused shifts of peaks corresponding to functional groups building glucose and amides II and III. Addition Au NPes to the U118 cells treated by X-ray radiation caused significant structural changes in all three amides and in glucose. In the Raman spectrum of U118 C@XcPt the absence of peak at 1292 cm^{-1} and shifts of peaks originating from amide II and lipids vibrations were visible, while addition Au NPes to this system additionally caused structural changes in glucose. In the Raman spectrum of U251 C@NPs, absence of peaks corresponding to amide II and CH vibrations from lipids, as well as shifts of peaks at 1362 cm^{-1}, 1455 cm^{-1}, were observed. Addition Au NPes to the U251 cell culture caused structural changes in the amide II and lipids vibrations visible as peaks at 1292 cm^{-1}, 1366 cm^{-1}, 1461 cm^{-1}, 2978 cm^{-1}, respectively. Cisplatin immobilized on the Au NPes surface caused, in the U251 cells, shifts of peaks corresponding to amides and lipids vibrations. In the Raman spectrum of U251 C@X absence of the peak at 1548 cm^{-1} and shifts of peaks at 988 cm^{-1}, 1350 cm^{-1}, 3324 cm^{-1}, were observed. Structural changes in all three amides vibrations, as well as in phenylalanine and lipids, were noticed in the Raman spectra of U251 C@XNPs, U251 C@XcPt and U251 C@XcPtNPs.

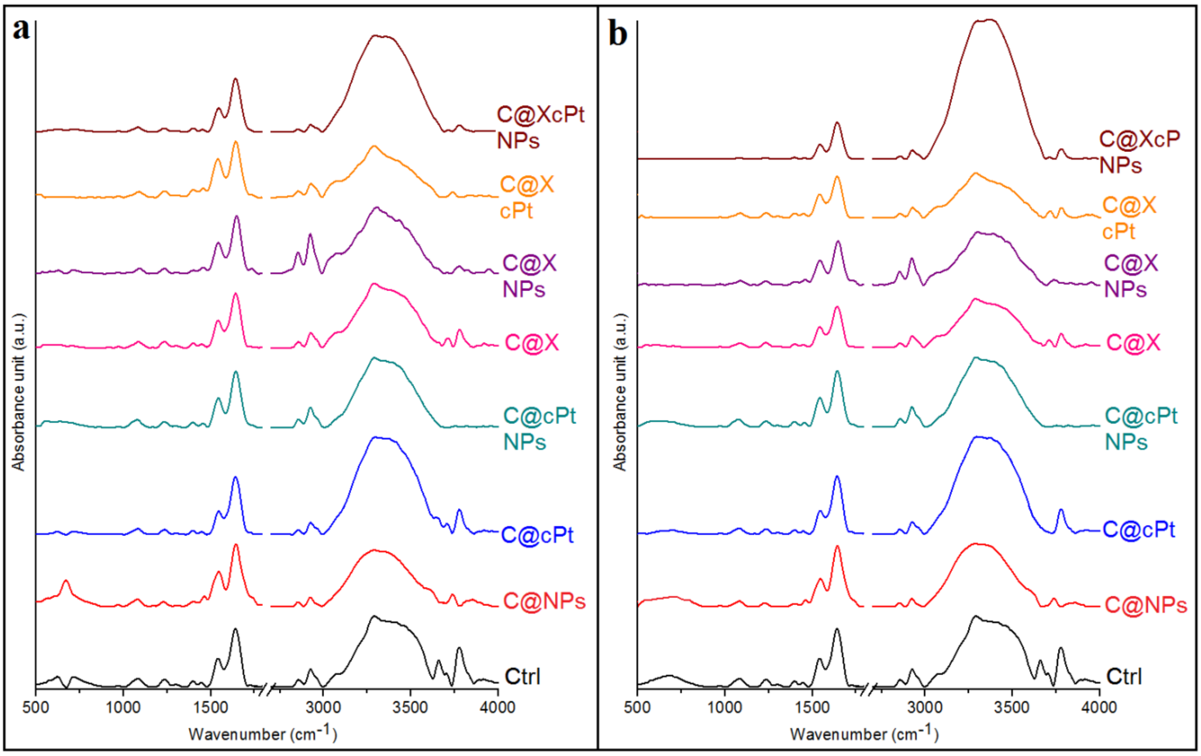

Figure S2. FTIR spectra of U118 (a) and U251 (b) cells: Ctrl (black spectrum); C@NPs (red spectrum); C@cPt (blue spectrum); C@cPtNPs (green spectrum); C@X (pink spectrum); C@XNPs (violet spectrum); C@XcPt (orange spectrum); C@XcPtNPs (brown spectrum).

The investigated samples were compared with the respective Ctrl. In the FTIR spectra of U118 C@NPs significant shifts of peaks at 1228 cm^{-1} and 1790 cm^{-1} were observed. Moreover, shifts of peaks corresponding to amide II and C=O vibrations, were visible in the FTIR spectrum of U118 C@cPt, while in the spectrum of U118 C@cPtNPs only a shift of the peak at 1745 cm^{-1} was noticed. Furthermore, X-ray radiation with Au NPs in U118 cells caused structural changes in the glycogen, lipids and proteins, while X-ray radiation of cells with cPt is responsible for the shifts of peaks

originating from functional groups building glycogen and lipids structure. In the FTIR spectrum of U118 C@XcPtNPs absence of the peak at 1735 cm^{-1} and shifts of the peaks at 1452 cm^{-1} and 1546 cm^{-1} were observed. In the cells only irradiated by X-rays, no changes in the peak positions were noticed. In the case of U251 cells cultured with Au NPes, shifts of the peaks corresponding to amides I and II, CH_2 and C=O groups from lipids, were visible, while cells cultured with cPt caused shifts of peaks originating from amide II and C=O group of lipids. In the FTIR spectrum of U251 C@cPtNPs, structural changes in glycol and lipids, were noticed, while in U251 C@XNPs changes in these chemical compounds and amide I, were observed. Cisplatin added to cells (C@cPt) subsequently irradiated by X-ray caused a shift of the peak corresponding to the C=O group from lipids, however, the addition of Au NPes to this sample caused changes in the nucleic acid (absence of peak at 971 cm^{-1}), lipids (absence of peaks at 1446 cm^{-1} and 1770 cm^{-1}) and proteins (shift of peak at 1540 cm^{-1}). The identified peaks for all the obtained FTIR and Raman spectra are presented in detail in Table 1.
Apart from DNA damage, cisplatin induces reactive oxygen species (ROS), which trigger cell death.[1] ROS have unpaired electrons therefore they are very reactive and act in response to proper functioning of biological macromolecules such as carbohydrates, lipids and proteins.[2] Moreover, the proteins are the major targets for biological oxidants. Therefore, oxidant formation is the major consequence of protein damage.[3] Moreover, as potential cytotoxic mechanisms of NPs, influence on the structure and functioning of cell membranes, metabolic process disorders by the formation of reactive oxygen species and negative effects on enzyme activity and DNA synthesis, are indicated.[4] The effect of NPs and cisplatin are visible in the Raman (Fig. 1) and FTIR (Fig. 2) spectra as absences or shifts of protein peaks. Moreover, cisplatin caused an increase of lipid peroxidation, which destroys the proper functioning of the cell membrane.[5] The changes in the lipids structure were also observed in the Raman and FTIR spectra.
X-rays are commonly used in anticancer therapies causing damaging of the DNA [6]. Moreover, it is known, that radiosensitization supported by gold nanoparticles is more effective, than X-ray therapy alone.[7] In this study, X-ray irradiation supported by Au NPes caused more visible structural changes in the cells, especially in the proteins, lipids and DNA, than X-ray irradiation alone. Furthermore, the most noticeable chemical changes are observed in the cell lines cultured with cPt@Au NPes and X-ray irradiated, which can be the synergistic effect of these two factors.

To resolve, whether it is sufficient to detect only the differences between the studied glioblastoma cell lines: U118 (Fig. 3a) and U251 (Fig. 3b), which were cultured with Au NPes, cPt or cPt@Au NPes and X-ray irradiated, PCA was used to analyze the obtained Raman and FTIR spectra.

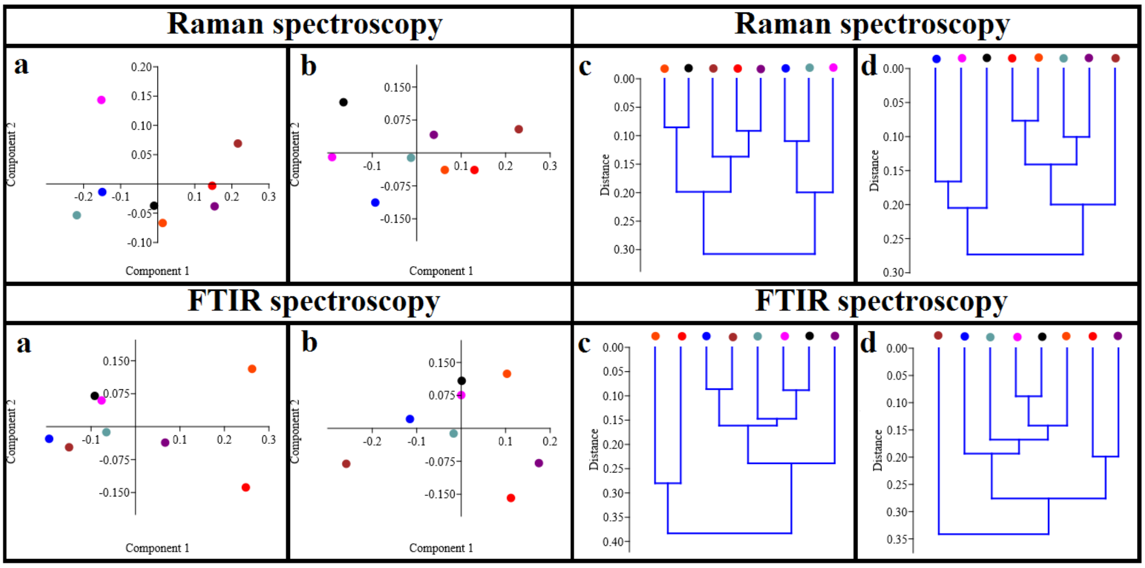

Figure S3. PCA analysis (a, b) and HCA analysis (c, d) of Raman and FTIR spectra of U118 (a, c) and U251 (b, d) cells: Ctrl (black dot); C@NPs (red dot); C@cPt (blue dot); C@cPtNPs (green dot); C@X (pink dot); C@XNPs (violet dot); C@XcPt (orange dot); C@XcPtNPs (brown dot).

The Raman PCA plot showed no separation of the control U118 cell lines and cells cultured with cPt and cPt@Au NPes, while in the case of the U251 cells, separation of other samples, was observed. Consequently, it can be concluded that the U251 cells could be more sensitive to the effects of drugs, X-rays and nanoparticles, as we showed in our previously research [7]. However, PCA analysis obtained from the FTIR spectra showed, that it is not possible to distinguish between the FTIR spectra of two analyzed control cell lines and cells treated by X-ray radiation. Moreover, the results obtained from the FTIR data are very similar when we compare the PCA analysis for U118 and U251 cell lines.

Cluster analysis performed from Raman and FTIR measurements (Fig. 3c, d) shows two main groups in the case of the U251 cell line (Raman data) and three in the U251 cell lines (FTIR data) and U118 (both Raman and FTIR data). However, depending on the method, the components in these groups are different. The most significant similarities between the control sample of U118 (Raman data) were observed for U118 C@XcPt. Moreover, similarities between U118 C@cPt and U118 C@cPtNPs, and between U118 C@NPs and U118 C@XNPs were observed. For the U251 cell lines, Raman similarities between C@cPt and C@X and between C@NPs and C@XcPt and between C@cPtNPs and C@XNPs, were noticed. It is very interesting that for HCA analysis obtained from the FTIR data, similarities between the control and the C@X samples for both U118 and U251 cell lines, were visible. Moreover, for U118 cell similarities between C@NPs and C@XcPt and between C@cPt and C@XcPtNPs, were noticed. FTIR spectroscopy showed similarities in the U251 cells between samples cultured with NPs and non-treated by X-ray.

Table S1. FTIR and Raman peaks positions in the analyzed samples with description of vibrations corresponding to the respective functional groups [1-14].

Raman spectroscopy																
**Ctrl**		**C@NPs**		**C@cPt**		**C@cPtNPs**		**C@X**		**C@XNPs**		**C@XcPt**		**C@XcPtNPs**		**Vibrations**
**U118**	**U251**	**U118**	**U251**	**U118**	**U251**	**U118**	**U251**	**U118**	**U251**	**U118**	**U251**	**U118**	**U251**	**U118**	**U251**	
879	873	888	880	875	879	886	877	875	870	885	871	885	871	886	880	C-H in-plane bending mode [8]
995	988	993	985	1005	990	-	985	998	1000	992	1005	991	-	1006	997	C-H in-plane bending mode of phenylalanine [8]
1089	1082	1080	1073	1091	1082	-	1081	1107	1084	1074	1078	1084	1073	1084	1086	Glucose [9], Triglycerides[10], C-C (lipid) [10]
71292	1262	-	1267	1262	1275	1268	1279	1279	1273	-	1277	-	1292	-	1265	Amide III [11]
1362	1350	1362	1367	1354	1366	1365	1369	1366	1364	1368	1361	1363	1366	1362	1364	CH_3/CH_2 twisting or bending mode of lipid/collagen [12,13]
1455	1449	1451	1483	1451	1461	1456	1459	1451	1456	1456	1458	1453	1472	1452	1453	Fatty acids [9], CH_2 (lipids and proteins [14]
1548	1577	1567	-	1580	1550	-	1549	1577	-	1578	-	1557	1555	1592	-	Amide II [11]
1655	1654	1662	1665	1661	1665	1661	1644	1653	1650	1668	1644	1659	1654	1659	1654	Amide I [11]
2934	2933	2936	-	2931	2978	2932	2938	2930	2938	2939	2926	2932	2897	2927	2978	CH band of lipids [11]
3397	3324	3329	3041	3404	3548	3405	3402	3397	3400	3393	3404	3406	3533	3537	3399	CH from cholesterol and cholesterol ester [14]
**FTIR spectroscopy**																
**Ctrl**		**C@NPs**		**C@cPt**		**C@cPtNPs**		**C@X**		**C@XNPs**		**C@XcPt**		**C@XcPtNPs**		**Vibrations**
**U118**	**U251**	**U118**	**U251**	**U118**	**U251**	**U118**	**U251**	**U118**	**U251**	**U118**	**U251**	**U118**	**U251**	**U118**	**U251**	
970	971	970	971	972	972	971	970	971	973	970	969	971	971	972	-	PO_3^{-2} group from DNA, RNA and phospholipids [15]
1083	1084	1082	1083	1082	1083	1079	1079	1086	1081	1092	1089	1088	1086	1083	1083	C-O group from glycogen [16]
1235	1235	1228	1227	1235	1235	1235	1234	1235	1236	1233	1234	1236	1236	1234	1234	Amide III [17]
1399	1399	1399	1397	1399	1399	1399	1399	1400	1400	1401	1399	1400	1400	1399	1399	CH_2 group from protein and Lipids [18,19]
1446	1446	1462	1462	1448	1448	1455	1454	1448	1448	1457	1458	1456	1449	1452	-	CH_2 group from cholesterol [19]
1540	1540	1544	1546	1546	1545	1543	1543	1543	1544	1544	1544	1540	1542	1546	1546	Amide II [17]
1641	1641	1644	1643	1642	1644	1643	1643	1642	1645	1648	1647	1643	1642	1642	1642	Amide I [17]

1765	1770	1790	1792	1756	1756	1745	1762	1765	1769	1735	1734	1742	1725	-	-	C=O group from lipids [19]
2856	2856	2853	2853	2855	2854	2857	2857	2855	2855	2853	2854	2854	2855	2855	2855	Symmetric stretching vibrations of CH_2 [19]
2925	2926	2924	2923	2926	2928	2924	2926	2927	2928	2924	2924	2927	2927	2926	2926	Asymmetric stretching vibrations of CH_2 [19]
3291	3291	3290	3286	3295	3291	3291	3295	3290	3292	3305	3302	3289	3290	3297	3369	OH from water [16]

References

1. Vitetta, L.; Linnane, A.W. Endocellular regulation of free radicals and hydrogen peroxide: key determinants of the inflammatory response. *Inflammopharmacology* **2014**, *22*, 69-72.
2. Ahmad, S.; Khan, H.; Shahab, U.; Rehman, S.; Rafi, Z.; Khan, M.Y.; Ansari, A.; Siddiqui, Z.; Ashaf, J.M.; Abdullah, S.M.S.; Habib, S.; Uddin, M. Protein oxidation: an overview of metabolism of Sulphur containing amino acid, cysteine. *Front. Biosci. (Schol. Ed.)* **2017**, *9*, 71-87.
3. Bystrzejewska-Piotrowska, G.; Golimowski, J.; Urban, P.L. Nanoparticles: their potential toxicity, waste and environmental management. *Waste Manag.* **2009**, *29*, 2587-2595.
4. Desoize, B. Cancer and metals and metal compounds: part I – carcinogenesis. *Crit. Rev. Oncol. Hematol.* **2002**, *42*, 1-3.
5. Harrison, L.B.; Chadha, M.; Hill, R.J.; Hu, K.; Shasha, D. Impact of tumor hypoxia and anemia on radiation therapy outcomes. *The Oncologist* **2002**, *7*, 492-508.
6. Ross, R.D.; Cole, L.E.; Tilley, J.M.R.; Roeder, R.K. Effects of functionalized gold nanoparticle size on X-ray attenuation and substrate binding affinity. *Chem. Mater.* **2014**, *26*, 1187-1194.
7. Bogdali-Suslik, A.M.; Rawojć, K.; Miszczyk, J.; Panek, A.; Woźniak, M.; Szewczyk, K.; Książek, T.; Bik-Multanowski, M.; Swakoń, J.; Komenda, W.; Mojżeszek, N.; Kajdrowicz, N.; Kopeć, R.; Olko, P. Influence of therapeutic proton beam on glioblastoma multiforme proliferation index – a preliminary study. *Acta Phys. Pol. A* **2020**, *137*.
8. Chan, J.W.; Taylor, D.S.; Zwerdling, T.; Lane, S.M.; Ihara, K.; Huser, T. Micro-Raman spectroscopy detects individual neoplastic and normal hematopoietic cells. *Biophys. J.* **2006**, *90*, 648–656.
9. Krafft, C.; Neudert, L.; Simat, T.; Salzer, R. Near infrared Raman spectra of human brain lipids. *Spectrochim. Acta A Mol. Biomol. Spectrosc*. **2005**, *61*, 1529–1535.
10. Silveira Jr, L.; Sathaiah, S.; Zangaro, R.A.; Pacheco, M.T.; Chavantes, M.C.; Pasqualucci, C.A. Correlation between near-infrared Raman spectroscopy and the histopathological analysis of atherosclerosis in human coronary arteries. *Lasers Surg. Med.* **2002**, *30*, 290–297.
11. Stone, N.; Kendall, C.; Smith, J.; Crow, P.; Barr, H. Raman spectroscopy for identification of cancers. *Faraday Discuss.* **2004**, *126*, 141–157.
12. Huang, Z.; McWilliams, A.; Lui, H.; McLean, D.I.; Lam, S.; Zeng, H. Near-infrared Raman spectroscopy for optical diagnosis of lung cancer. *Int. J. Cancer* **2003**, *107*, 1047–1052.
13. Malini, R.; Venkatakrishna, K.; Kurien, J.; Pai, K.M.; Rao, L.; Kartha, V.B.; Krishna, C.M. Discrimination of normal, inflammatory, premalignant, and malignant oral tissue: a Raman spectroscopy study. *Biopolymers* **2006**, *81*, 179–193.
14. Stremersch, S.; Marro, M.; Pinchasik, B.E.; Baatsen, P.; Hendrix, A.; De Smedt, S.C.; Loza-Alvarez, P.; Skirtach, A.G.; Raemdonck, K.; Braeckmans, K. Identification of individual exosome-like vesicle by surface enhanced Raman spectroscopy. *Small* **2016**, *12,* 3292–3301.
15. Maziak, D.E.; Do, M.T.; Shamji, F.M.; Sundaresan, S.R.; Perkins, D.G.; Wong, P.T. Fourier-transform infrared spectroscopic study of characteristic molecular structure in cancer cells of esophagus: an exploratory study. *Cancer Detect Prev.* **2007**, *31*, 244–253.
16. Krishna, C.M.; Sockalingum, G.D.; Bhat, R.A.; Venteo, L.; Kushtagi, P.; Pluot, M.; Manfait, M. FTIR and Raman microspectroscopy of normal, benign, and malignant formalin-fixed ovarian tissues. *Anal Bioanal Chem.* **2007**, *387*, 1649–1656.
17. Movasaghi, Z.; Rehman, S.; ur Rehman, I. Fourier transform infrared (FTIR) spectroscopy of biological tissues. *Appl. Spectrosc. Rev.* **2008**, *43*, 134–179.
18. Elmi, F.; Movaghar, A.F.; Elmi, M.M.; Alinezhad, H.; Nikbakhsh, N. Application of FT-IR spectroscopy on breast cancer serum analysis. *Spectrochim Acta A Mol Biomol Spectrosc.* **2017**, *187*, 87–91.
19. Yano, K.; Ohoskima, S.; Gotou, Y.; Kumaido, K.; Moriguchi, T.; Katayama, H. Direct measurement of human lung cancerous and noncanceroustissues by fourier transform infrared microscopy: can an infrared microscope be used as a clinical tool? *Anal Biochem.* **2000**, *287*, 218–225.
